# Effect of Using Steel Bar Reinforcement on Concrete Quality by Ultrasonic Pulse Velocity Measurements

**DOI:** 10.3390/ma15134565

**Published:** 2022-06-29

**Authors:** Ominda Nanayakkara, Hadee Mohammed Najm, Mohanad Muayad Sabri Sabri

**Affiliations:** 1 Department of Civil Engineering, Xi’an Jiaotong-Liverpool University, Suzhou 215000, China; 2 Department of Civil engineering, Zakir Husain Engineering College, Aligarh Muslim University, Aligarh 202002, India; gk4071@myamu.ac.in; 3 Peter the Great St. Petersburg Polytechnic University, 195251 St. Petersburg, Russia; mohanad.m.sabri@gmail.com

**Keywords:** concrete, steel reinforcement, compressive strength, non-destructive test, ultrasonic pulse velocity

## Abstract

Non-destructive tests (NDTs) represent one of the solutions that aid engineers in evaluating the strength of materials. However, the results obtained using such tests are still questionable as they may be affected by different factors. One of these factors is the presence of steel reinforcement in concrete. An experimental investigation is presented in this study to investigate the effect of the single reinforcement steel bar on ultrasonic pulse velocity (UPV). Seven concrete beams, one containing no steel and the other six beams containing varying bar diameter and cover thicknesses, were tested. UPV measurements were obtained using the indirect method and then modified to eliminate the effect of the steel bar. To provide the scientific evidence to give a reliable and reasonable solution, a statistical analysis was also conducted. The results show that a large bar diameter and a small cover thickness significantly influence the measured UPV. Measured UPV with a spacing between transducers up to 500 mm can effectively be used to predict the compressive strength of concrete after the modification of the initial UPV.

## 1. Introduction

Concrete testing is a crucial procedure in a construction project or laboratory experiment. It is typically time-consuming and intractable, especially in a place where no samples can be taken from existing structures to be tested. At the same time, the non-destructive test (NDT) is becoming more popular in the built environment around the world. It is used to determine the integrity of a material, a component of the structure, or quantitatively measure some characteristic of an object. The NDT is a highly valuable technique that saves both material and financial resources in product evaluation, overhauling, and research.

To determine the compressive strength of concrete, destructive coring tests, as well as NDT methods, such as the rebound hammer, ultrasonic tests, and radar, are widely used [1]. However, because each testing method has advantages and disadvantages, continuous efforts are still being made to improve the accuracy and reliability of these methods [2,3].

Test methods are also categorised into penetration and nonpenetration modes. Because concrete typically exhibits variations in its material property even within a single member, concrete strengths are better obtained via penetration mode tests, such as in ultrasonic testing by passing through the entire cross-section of a member rather than part of it, as in the nonpenetration or reflection mode.

An ultrasonic pulse velocity test is an in situ, non-destructive test to determine the homogeneity of the concrete. In this test, the strength of concrete can be assessed by measuring the ultrasonic pulse velocity (UPV) that passes through a concrete structure [4]. In addition, it can also detect the presence of internal imperfections, defects, cracks, and voids that may occur due to the cement hydration, frost, or fire damage in concrete. There are several factors influencing UPV results, such as temperature, moisture content, path length, shape and size of the specimen, steel fiber, and reinforcing bars [5].

The experimental apparatus for measuring UPV is advanced and convenient. The arrangement of transducers is also flexible and varies with the different situations. However, ultrasonic waves are known to be affected by the presence of embedded steel inside the concrete. Thus, it is necessary to quantitatively investigate the significance of the effect of steel.

When dealing with the effect of steel in concrete on the estimation of the compressive strength of concrete, two scenarios need to be considered. First, it has been reported that when an ultrasonic wave crosses the steel bars in a direction that cuts through the cross-sectional areas, the summation of the cross-sectional areas of steel causes the wave velocity to increase [6,7]. Second, it has been reported that when the wave travels through the steel bars in a direction parallel to the length of the bars, the wave velocity also increases [7,8]. Based on these reports, the codes of some countries recommend that testing methods apply correction factors [9,10].

Currently, steel-reinforced concrete structures are used extensively worldwide, but the presence of reinforcement causes considerable uncertainties due to the higher velocity of pulses in steel [11]. It is misleading that the first pulse to arrive at the receiving transducer passes through partly both in concrete and in steel, thereby giving an inaccurate result of concrete quality. Although the British Standard suggests a series of calculations and correlations if reinforcement exists under the situation of ‘direct transmission’, it still recommends that ‘wherever possible, measurements should be conducted in such a way that steel does not lie in or close to the direct path between transducers’ [12].

In addition, in concrete it is always the case that that only one surface is appropriate for performing the ultrasonic pulse test. For instance: in a column with only one face exposed to the inner space, it is not always possible to apply the direct transmission. In order to obtain the quality of the concrete, indirect transmission is the only method. Therefore, the problem was raised, and this project is going to solve the contradiction of avoiding the use of the ultrasonic pulse velocity method in concrete structures if a single reinforced bar is present under the condition of indirect transmission. One of the methods of testing both steel and plastic bars is the self-excited acoustical system [13,14].

The present study aimed to investigate the quality of the concrete with the presence of a single reinforced steel bar by using an indirect transmission. So, it looks at the effect of the reinforcing steel bar diameter and concrete cover on the pulse velocity when it crosses the reinforced concrete medium. Three sizes of bar diameter and four concrete cover ranges were considered, so seven beams and twelve cubes were cast, namely, C20D20, C40D20, C60D12, C60D20, C60D32, and C80D20. By comparing beams C20D20, C40D20, C60D20, and C80D20, the effect of the concrete cover was able to be identified. Similarly, by comparing the beams C60D12, C60D20, and C60D32, the effect of the bar size was able to be investigated.

It has been observed through the literature that there are existing experimental works to evaluate the concrete quality by UPV Measurements [15,16,17]. However, there is no available experimental work to evaluate the compressive strength of concrete with the effect of different diameters of steel bar reinforcement and different concrete covers by UPV measurements.

## 2. Experimental Setup and Theory

### 2.1. Experimental Plan

To achieve the objectives of the present study, different parts have been conducted:

i. Identify the existence of the steel bar in the concrete: The information about concrete in a construction structure is always unknown, including whether the reinforcement exists or not. If the concrete is not reinforced, the analysis will be meaningless.
ii. Analyse the pulse velocity with the steel bar existing: In this part, the calculation is performed to avoid the influence of the steel bar.
iii. Perform a statistical analysis: This is to provide the scientific evidence to give a reliable and reasonable solution. On the basics of the statistical analysis, recommendations can be made.

Previous studies have found that many factors influence UPV if the steel bar exists, such as concrete cover, bar size, corrosion, the bond between steel bar and concrete, the direction of steel bar, and the number of steel bars. However, in this study, only a single reinforcement is taken into consideration, and the steel bar is located at the middle line of a lateral cross-section. A deformed reinforcing steel bar with a grade of HRB500 (ASTM) was used in the study. Considering varying concrete cover thickness and the bar diameter, seven beams and twelve cubes were cast, as shown in Table 1. Consequently, the results of each beam can be compared with the plain concrete. By comparing beams C20D20, C40D20, C60D20, and C80D20, the effect due to the concrete cover can be identified. Similarly, by comparing the beams C60D12, C60D20, and C60D32, the effect due to the bar size can be investigated.

The arrangement of the emitter and receiver transducers varies due to the length of the beam. Thus, a longer length of the beam is preferred. The cross-section has a limitation according to the British Standard [9]. The short pulse of vibration is independent of the size and shape of the specimen unless the lateral dimension has a certain minimum value. If the minimum dimension of a cross-section is less than the wavelength, the mode of propagation will change. Therefore, the measured UPV will be different and inaccurate. As a result, a concrete beam with a width of 130 mm, depth of 165 mm, and length of 1200 mm was designed. In addition, 12 cubes with a side length of 150 mm were also designed to be cast at the same time to ensure the consistency of concrete. These cubes were tested for compressive strength both at 14 and 28 days.

### 2.2. Concrete Mix Design

**Figure 1:**
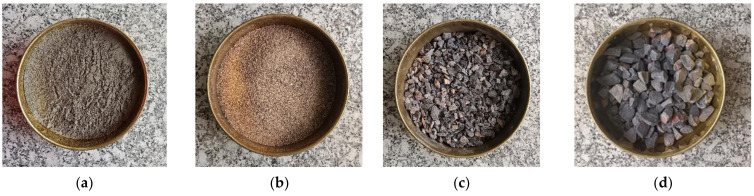
(**a**) Commercial cement; (**b**) river sand; (**c**) natural coarse aggregates (NCA)—10 mm; (**d**) natural coarse aggregates (NCA)—20 mm.

The aim of having a concrete beam is to investigate the effect of the presence of a steel bar on the UPV. Thus, the concrete does not need a specific compressive strength. The concrete mix design is based on a water/cement ratio of 0.55 as the main factor, which directly influences the concrete strength. The ratio between cement and aggregates also has strong effects on the material capacity [18]. According to the design of normal concrete mixes, the concrete beam was designed to have a characteristic strength of 30 N/mm^2^ at 28 days. The cement strength class was 42.5 N. The maximum coarse and fine aggregate had a size of 20 mm and 5 mm, and the type was crushed Basalt stone and river sand, respectively. The slump was in the range of 30–60 mm. Table 2 and Figure 1 shows the concrete materials and mix composition.

### 2.3. UPV Equipment and Operation

**Figure 2:**
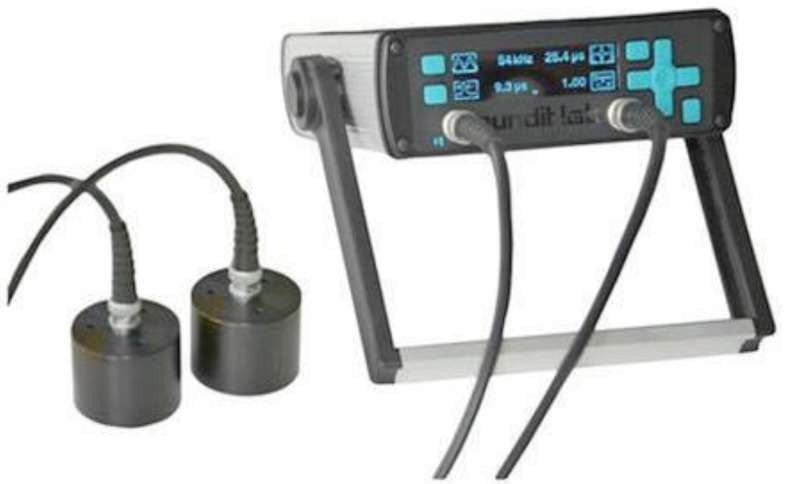
Photo of Pundit Lab+.

The Pundit Lab+ offered by Proceq, as shown in Figure 2, was applied in this project to conduct ultrasonic testing. It is a UPV test instrument designed primarily for operation in laboratories that supports all traditional UPV test modes. The measuring range is up to 15 m depending on the concrete quality [19]. The nominal transducer frequency is from 24 to 500 kHz. The measuring resolution is 0.1 microseconds.

The distance (path length) between the transducers should be measured as accurately as possible to ensure the correct path length of the pulse. Adequate acoustic coupling of the transducers to the surface is also important to ensure transducer sensitivity. A thin layer of coupling agent should be applied to the transducer and the test surface [20]. In some cases, if the testing surface is rough, polishing or smoothing the surface is necessary. The distance between transducers needs to be set so that the test can be started.

### 2.4. UPV of Concrete at the Presence of Steel Bar

#### 2.4.1. Methodology

**Figure 3:**
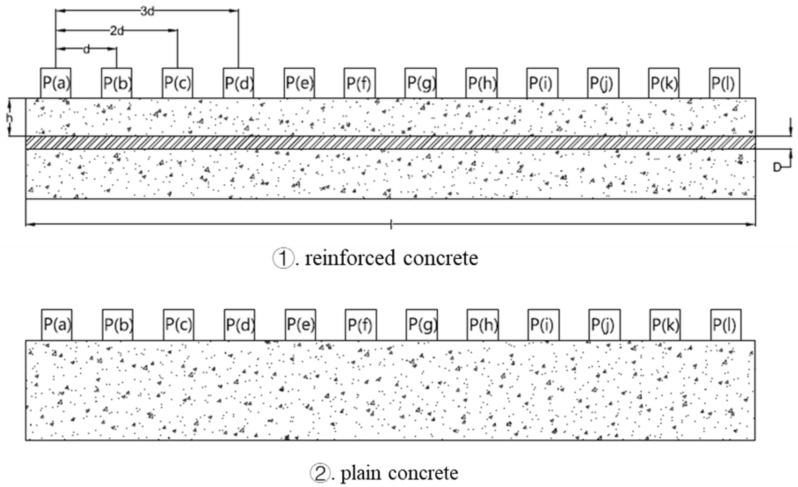
The arrangement of transducers.

There are 12 positions where the transducers are placed, as shown in Figure 3. The transducers from left to right are labelled as P(a), P(b), P(c), …, and P(l). The transit time was measured by placing the emitter on P(a) and the receiver on all the other points, starting from point P(b). In this case, the distances between transducers change from 100 mm to 1100 mm, where d=100mm was taken as the shortest distance between transducers. The transit time taken is also different due to varying lengths and the presence of the steel bar at different cover depths. Then, the procedure was repeated by placing the emitter at point P(b) and the receiver on all other right-side points. In this case, the distance between transducers changes from 100 mm to 1000 mm. Finally, the location of the emitter is changed from point P(a) to P(k), and 66 data points were obtained for each beam.

#### 2.4.2. Theory

Steel bar affects the transit time between transducers because the UPV in the steel bar is faster than that of concrete. If there is a path that makes the wave reach the receiver faster, it emphasises the influence of the steel bar. This can be observed by comparing the UPV results of the concrete beam with and without a steel bar. Therefore, this section aims to solve the UPV of concrete ($V_{c}$) using measurement of reinforced concrete. The pulse velocity (v) is equal to the ratio of the distance between transducers (L) and the time taken by the pulse to travel (T), as given in Equation (1) [9].

(1) $$v=\frac{L}{T}$$

**Figure 4:**
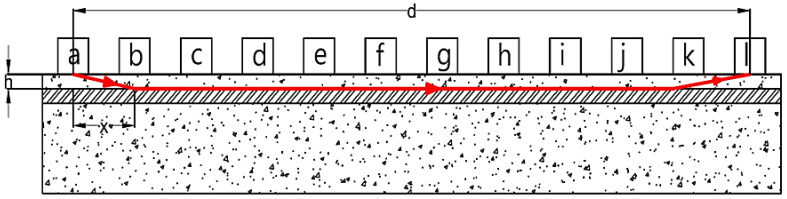
The pathway of the wave under the presence of a steel bar.

Assume that the transducers are placed on the points P(a), P(l), and the distance between two pints is d, as shown in Figure 4. In this situation, the red colour line shows the fastest wave as the wave travels through the steel bar faster. The wave passes through part of concrete and part of steel bar at the same time. The unknowns are the concrete cover (h), deviation (x), and wave velocity both in concrete ($V_{c}$) and steel bar ($V_{s}$).

The transit time will be the addition of the time passing through partly within concrete ($t_{c}$) and partly in the steel bar ($t_{s}$).

(2) $$t=t_{s}+t_{c}$$

Further substituting the travel distance within concrete ($L_{c}$) and within steel ($L_{s}$), the total travel is given by,

(3) $$t=\frac{L_{s}}{V_{s}}+\frac{L_{c}}{V_{c}}$$

Using the horizontal travel distance (i.e., deviation) and the vertical travel distance (i.e., cover thickness) within the concrete is x and h, respectively, the transmit time is given by Equation (4). The distance between transducers is d. The transit time (t) is a function of d, x, $V_{s}$, $V_{c}$, and h.

(4) $$t=\frac{d-2x}{V_{s}}+\frac{2\sqrt{x^{2}+h^{2}}}{V_{c}}$$

(5) $$t=f\left(d,\;x,V_{s},V_{c},h\right)$$

Once the derivative of t due to the deviation x is taken, the pathway that makes travel time minimum can be obtained. Thus, the derivative of t due to x is given by,

(6) $$\frac{dt}{dx}=-\frac{2}{V_{s}}+\frac{2x}{\left(\sqrt{h^{2}+x^{2}}\right)V_{c}}$$

Let $\frac{dt}{dx}=0$, the deviation x is given by,

(7) $$x=\pm \frac{V_{c}h}{\left(\sqrt{V_{s}^{2}-V_{c}^{2}}\right)}$$

The negative value of x is ignored because x is the deviation that makes times faster rather than slower. Interestingly, the pathway is not related to the distance between transducers, i.e., d, in other words, regardless of the locations of transducers, the theoretical result is the same. Substituting the value x into t,

(8) $$t=\frac{d}{V_{s}}+\frac{2h\sqrt{V_{s}^{2}-V_{c}^{2}}}{V_{s}V_{c}}$$

In an infinite medium, the pulse velocity of steel is taken as 5900 m/s. It is known that the pulse velocity in steel bars decreases by a certain value if considered a concrete–steel medium. The pulse velocity of steel in a concrete medium with a bar diameter of D is given by the previous study [21],

(9) $$V_{s}=5900-\frac{10.4\left(5900-V_{c}\right)}{D}$$

Substituting the $V_{s}$ in Equation (9) into the Equation (8), the equation of t can be solved as,

(10) $$t=\frac{D}{\gamma \sqrt{∆}}\left(5d\sqrt{∆}+\frac{\left(12D-123+21V_{c}\right)h\gamma }{V_{c}D}-10hV_{c}D\right)$$

Where ∆ and γ are expressions given by Equations (11) and (12), aiming to simplify the equation for t, and they do not have any physical meaning.

(11) $$∆=108V_{c}^{2}-V_{c}^{2}D^{2}+35D^{2}-724D+123DV_{c}+3765-1276V_{c}$$

(12) $$\gamma =30D-307+52V_{c}^{2}$$

According to the Equation (10), the transit time (t) is a function of concrete cover (h), steel bar diameter (D), the distance between transducers (d), and the pulse velocity in concrete ($V_{c}$). The h and D should be fixed values in a specific beam so that $V_{c}$ can be determined using the measured transit time for a given d as shown in Equation (13).

(13) $$t=f\left(V_{c}\right)$$

Each UPV measurement provides data on transit time (t) and the distance between transducers (d). From the obtained data of t and d, a MATLAB code was used to convert the measured transit time into pulse velocity concrete. In one reinforced concrete beam, the d changes from 100 mm to 1100 mm at an interval of 100 mm. The 66 measured data points in one beam were used to obtain 66 numbers of $V_{c}$ values, which are called modified pulse velocity. By repeating the procedure, modified pulse velocity can be obtained for all the concrete beams with embedded reinforcement.

### 2.5. Statistic Analysis of UPV

The average value of the apparent and modified pulse velocity was used to study the influence of the steel bar. However, the average value cannot represent the feature of each value. In total, 66 data points of the transit time were measured in one single beam. After the application of the theory, 66 modified UPVs in concrete were be obtained. The implementation of summary statistics is an important method in the undertaking of any statistical analysis.

The data were to be interpreted by setting the frequency and relative frequency. This has the advantage that the ratio between each range can be easily observed. In this investigation, the modified UPV is shown individually and divided into different groups with the same interval of 200 m/s. Moreover, the mean, median, and extreme data points are calculated. Sometimes, the median value is a more representative value than the mean value if the standard deviation is large [22].

## 3. Results and Discussions

### 3.1. Results by Existence of Steel Bar in Concrete

The experimental data for concrete beams were obtained at the age of 120 days. The measured transit time was converted to the pulse velocity, taking the relevant spacing between transducers into account. Calculated UPV, together with the average and the standard deviation for each spacing of the concrete beam without a steel bar (plain beam), is shown in Table 3.

**Figure 5:**
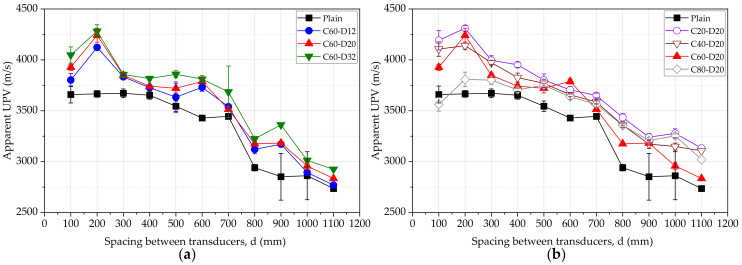
UPV of beams, (**a**) varying bar diameter, (**b**) varying cover thickness.

Theoretically, the UPV in the plain concrete is expected to be equal regardless of the location of transducers along the beam. However, practically, it can be seen that the average value of the UPV has a decreasing tendency with increased spacing between transducers, as shown in Figure 5. UPV of beams with changing bar diameter and changing cover thickness is shown in Figure 5a,b, respectively. It can be seen that the pulse velocity in the plain concrete is similar up to the spacing of 400 mm, and the UPV continuously decreases thereafter. All the beams with a steel bar show a higher UPV in comparison to the plain beam. This is due to the existence of the steel and higher pulse velocity within the steel. To uncover the most accurate UPV, the direct transmission was applied, and UPV was measured as 3669 m/s. This result matched the measured average UPV of 3663 m/s for locations from 100 mm to 400 mm. Therefore, the spacing up to 400 mm provided accurate results similar to the direct test. However, the presence of the steel bar significantly affected the measured data and cannot be directly used to interpret the properties of concrete.

**Figure 6:**
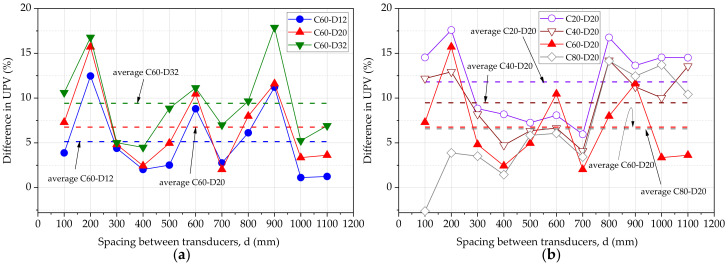
% change in UPV (**a**) varying bar diameter (**b**) varying cover thickness.

The percentage change of the UPV for the plain concrete beam does not show a unique relationship with the spacing between transducers, as shown in Figure 5. The percentage change in the UPV increases with the diameter of the steel bar and decreases with the cover thickness of steel, as shown in Figure 6a,b, respectively. The average percentage change of UPV is 5.14%, 6.75%, and 9.41% for beams C60D12, C60D20, and C60D32, respectively. This is attributed to the higher pulse velocity through the steel, and the larger bar diameter increases the wave velocity. Average percentage change of UPV is 11.81%, 9.47%, 6.75%, and 6.57% for beams C20D20, C40D20, C60D20, and C80D20, respectively. This observation is due to the deviation of the steel bar from the measuring surface of concrete, reducing the pulse velocity as the steel bar affects less for the wave transit.

**Figure 7:**
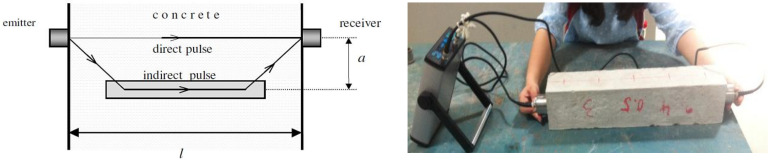
Direct test in reinforced concrete.

According to the British Standard [9], the energy is lost because the faces of transducers are not opposite in indirect transmission. Consequently, the lost energy leads to an unstable signal at the receiver, thereby influencing the accuracy of the transit time. Furthermore, the average results seem to be irregular, and it is not possible to find the relationship between the lost energy due to the increasing distance of transducers and decreased pulse velocity. At the same time, the direct transmission cannot be applied to obtain the pulse velocity in beams because the deviation from the transducers to the steel bars, i.e., a, requires approximately 1/6 to 1/8 of l, as shown in Figure 7. According to this study, the deviation needs to be at least 150 mm, which was not sufficient to avoid the effect of the steel bar. To identify the existence of steel bars in the concrete and to determine the UPV of plain concrete when the steel bar exists, the two results of plain and reinforced concrete need to be further compared.

### 3.2. Analysed Results of Pulse Velocity

According to Ndagi, A. et al. [23], “The pulse velocity is not affected in any way by the geometry or shape of the concrete material being tested. However, it is affected by factors such as cement type, aggregate type and size, water-cement ratio, the distance between transducers, admixtures, positioning of the transducers and concrete age”.

The modified pulse velocity for the concrete medium using the measured pulse velocity of concrete beams with steel was calculated based on Equations (10)–(12). Modified pulse velocity is not applicable for the plain concrete beam as the beam does not have a steel bar.

**Figure 8:**
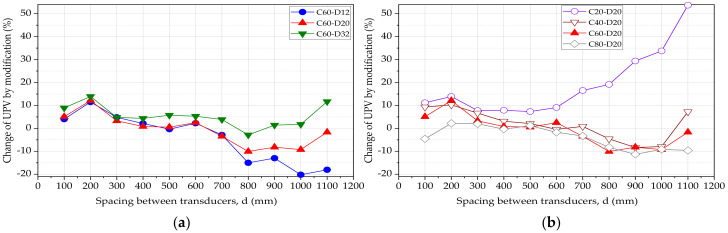
Change of UPV by modification (**a**) varying bar diameter (**b**) varying cover thickness.

Figure 8 shows modified UPV results for the beams with varying bar diameters (Figure 8a) and varying concrete cover thickness (Figure 8b). By comparison with the apparent pulse velocity shown in Figure 5, there is a distinct difference after the modification. It can be observed from the similarities that both modified and apparent pulse velocities have a decreasing trend as the distance between transducers increases. The modified pulse velocity is lower than the apparent pulse velocity at some spacing values while higher in other spacing values. This observation is within the expectations since the pulse wave passes partly through the steel bar at the first measurement. For the beams with a constant cover of 60 mm, the % change in the UPV is approximately ±15%, as in Figure 8a. A larger bar diameter tends to decrease the modified UPV in comparison to the measured UPV. For concrete beams with a constant bar diameter of 20 mm, the change in UPV is approximately within the range of ±10%, as in Figure 8b. Only the beam with a cover thickness of 20 mm shows a significant variation after 600 mm spacing.

**Figure 9:**
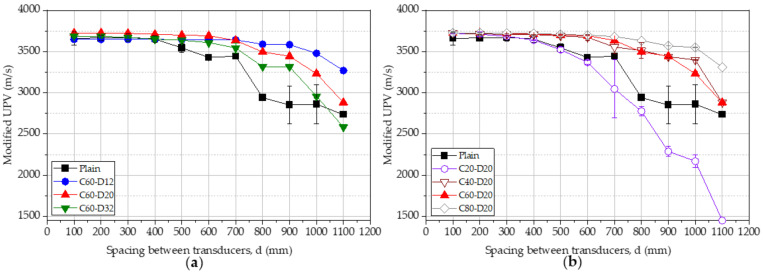
Modified UPV of beams, (**a**) varying bar diameter, (**b**) varying cover thickness.

In all the cases, the modified UPV results have a similar trend with a plain concrete beam from 100 mm to 400 mm spacing between transducers, as shown in Figure 9a,b. For this range, the difference between the UPV with the plain concrete beam is less than 2.00%. For the spacing up to 500 mm, all the modified UPV results are within the range of 5.00% for the plain concrete beam. The other values which still deviate from the UPV results of the plain concrete are due to the lost energy because the derived equation is not able to consider the lost energy effect. The sensitivity of the equations to modify UPV values becomes lower when the transducers are placed more than 500 mm. With the increase of the concrete cover, the modification becomes weaker if the spacing between transducers is over 600 mm. There is no doubt that a 20 mm concrete cover should have the most significant influence on the pulse wave because the steel bar is the closest one to the face of transducers. However, the error from the concrete cover that is greater than 20 mm can be omitted once the distance between the transducer is lower than 500 mm. Most researchers suggested and implemented in the indirect transmission that the distance between transducers is always smaller than 600 mm [24,25,26]. Therefore, 100 mm to 500 mm of the distance between transducers can still provide a good estimation of the quality of the concrete.

### 3.3. Compressive Strength

**Figure 10:**
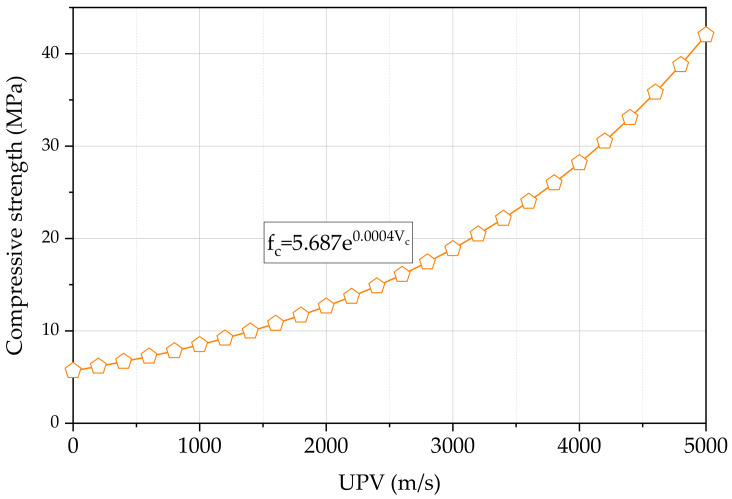
Fitting of measured UPV and compressive strength.

The Pundit Lab guideline provides a method to estimate the compressive strength of concrete using the UPV value and the rebound number as shown in Equation (14) [20]. In this equation, V and S are pulse velocity (m/s) and rebound hammer number, respectively, with a, b, and c constants. The compressive strength can also be presented as an exponential relationship with pulse velocity as shown in Equation (15) [27]. In this equation, V is the pulse velocity (m/s) with a and b constants. Similar equations have been developed in other studies for lightweight concrete [28] and rubberised concrete [29]. This study adopts the development of an exponential equation similar to Equation (15) to find the relationship between the compressive strength and the UPV. Six standard concrete cubes were tested for compressive strength with the size of 150 mm at the age of 28 days, as shown in Table 4. It can be reasonably assumed that the concrete used in this study gives similar compressive strength at 28 days and 120 days of UPV measurements of concrete beams. In addition, for the compressive strength, six average data of UPV measured using the same concrete cubes were also required. Based on six pairs of data, Equation (16) was developed. $f_{c}$ (MPa) is the measured compressive strength of concrete. Figure 10 shows the variation of the compressive strength against the UPV as predicted by Equation (16). Equation (16) gives a compressive strength of 30.5 MPa for a pulse velocity of 4200 m/s, while Equation (15) with a=0.00055, b=0.0025, and V=4200 m/s gives a compressive strength of 19.9 MPa for a coarse aggregate content of 1100 kg/m^3^.

(14) $$f_{c}=aV^{b}S^{c}$$

(15) $$f_{c}=ae^{bV}$$

(16) $$f_{c}=5.687e^{0.0004V_{c}}$$

Table 5 summarises the average of the modified UPV values for the transducer spacing from 100 mm to 500 mm. It was previously shown that the modified UPV of concrete with a steel bar is in good agreement with the measured UPV of plain concrete for the transducer spacing from 100 mm to 500 mm. Thus, the modified UPV, ranging from 100 mm to 500 mm, can be used to determine the average UPV and hence to determine the actual compressive strength of concrete using Equation (14). According to Table 4, the actual average compressive strength is 27.0 MPa, considering six cubes. This average can be further accurately calculated by ignoring the compressive strength of Cube 6. As a result, is slightly deviated from all other five cubes. In that condition, the average compressive strength is 26.1 MPa. Therefore, according to Table 5, the compressive strength predicted by the modified UPV is in good agreement with measured compressive strength.

### 3.4. Analysis of Modified UPV by Statistical Method

In the previous section, the average value with the standard deviation of the apparent and modified UPV was used to study the influence of the steel bar on the UPV measurements. However, the average value cannot represent the feature of each value when there are 66 data points of the transit time measured in one single beam with different spacing between transducers. It is difficult to interpret a single data point on its own. The implementation of summary statistics is an important method in the undertaking of any statistical analysis.

**Figure 11:**
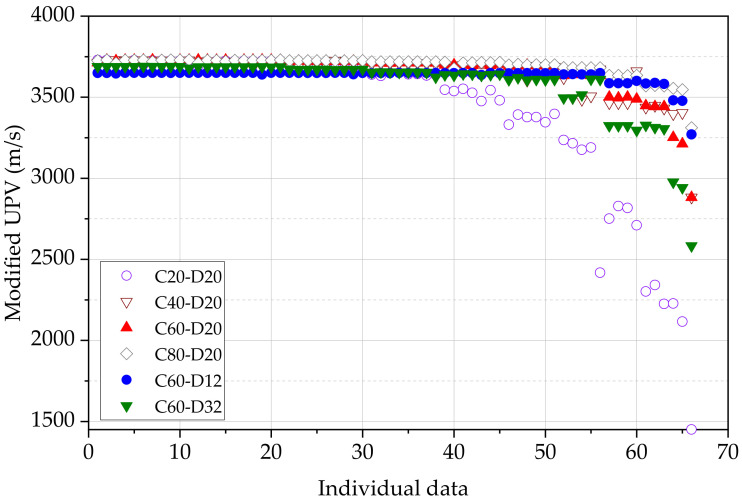
All data points of modified pulse velocity.

Figure 11 shows the variation of the 66 modified UPV data points for all the beams. Each data point represents one of the tests with traducers located at designated points. This variation is the same as the variation presented in Figure 9b; however, Figure 11 shows all the data points. Some significant differences between each value still exist as a result of initially measured apparent UPV. The beam of C20D20 starts to show the fluctuation of modified UPV from the 39th data point, which means that the data are reliable and can be used in compressive strength calculations until the transducer spacing of 500 mm. All other beams generally start to fluctuate of modified UPV from the 52nd data point, which means that the data are reliable until 600 mm of transducer spacing. The highest reliability is shown by the beam C80D20 due to the highest cover thickness of concrete and minimum influence of the variation of the cover thickness. The beam C20D20 has a concrete cover thickness of 20 mm, and a small variation in the concrete cover could significantly vary the UPV measurement. This kind of inconsistency in the cover thickness is highly possible in a long concrete beam with a small concrete cover thickness.

Table 6 summarises the frequency of the modified UPV for the full range of velocity. UPV results were grouped into bins, starting from 1400 m/s to 3800 m/s with a range of 200 m/s. There are no modified UPV data observed below 1200 m/s and above 3800 m/s in this study, although there are some apparent UPV measurements outside this range. The calculated maximum and minimum UPV values are 3729 m/s and 1451 m/s, respectively. The average value of modified UPV is 3398 m/s, which is not reliable as a result of available extreme data points. The median value of the modified UPV is 3642 m/s, which is a good reflection in terms of the whole sample.

Considering the full set of frequency data presented in Table 6, the UPV in the range of 3600 m/s to 3800 m/s can be identified with the highest frequency in each beam. Except for the beam C20D20, all the beams have a relative frequency above 80% for the UPV range of 3600 m/s to 3800 m/s. This result further confirms the acceptance of the modified UPV to predict the compressive strength of concrete medium in reinforced concrete. The compressive strength from Equation (16) is 24.0 MPa and 26.0 MPa for the modified UPV of 3600 m/s and 3800 m/s, respectively. As also explained in Section 3.3, this observation satisfies the idea that the actual compressive strength can be accurately predicted using modified UPV.

## 4. Conclusions

This study was carried out to investigate the effect of steel bars in concrete on the UPV measurements. This study proposed a method to determine the UPV applicable for plain concrete in reinforced concrete by considering experimental evidence with plain concrete, varying cover thickness, and varying steel bar diameter. Based on the study and analysis, the following conclusions can be drawn.

1. The existence of steel, mainly as reinforcement in the same direction of indirect UPV measurement, can significantly influence the measured UPV.
2. Large bar diameter and small concrete cover thickness increase the UPV measurement using the indirect method.
3. The measured indirect UPV data, which was influenced by the presence of steel, can be converted to a modified UPV using a mathematical approach.
4. The modified UPV of concrete, with a cover thickness of 20 mm to 80 mm, can predict the UPV of plain concrete with an accuracy of 95% if the transducer’s spacing is between 100 mm and 500 mm, using indirect measurements. Transducer spacing can be further increased to 600 mm or 700 mm if the concrete cover thickness increases to 40 mm and 80 mm, respectively.
5. Modified UPV accurately predicts the compressive strength of plain concrete. This was achieved using existing databases on UPV data for different types of concrete.
6. This method of determining the indirect UPV of plain concrete in reinforced concrete elements should be carefully conducted. The results of this study do not support the situations with more than one steel bar in the longitudinal direction with the measurement, as more steel bars can influence the accuracy of the data. However, the steel bars in the lateral direction of the measurement will have minimal influence on the measured data.

## Figures and Tables

**Table 1:** Concrete beams casting plan.

	C20D20	C40D20	C60D12	C60D20	C60D32	C80D20	Plain
Concrete cover, mm	20	40	60	60	60	80	Plain concrete (No steel bar)
Bar size, mm	20	20	12	20	32	20	

**Table 2:** Concrete mix design.

Materials	Cement	Water	Fine Aggregate	Coarse Aggregate
Quantities (kg/m^3^)	345	202	700	1162

**Table 3:** The apparent UPV in plain concrete.

Spacing between Transducers (mm)	Emitter Location & Apparent Pulse Velocity (m/s)												
	P(a)	P(b)	P(c)	P(d)	P(e)	P(f)	P(g)	P(h)	P(i)	P(j)	P(k)	Average	Standard Deviation
100	3533	3717	3649	3597	3663	3546	3649	3684	3703	3690	3831	3660	83
200	3696	3597	3683	3663	3669	3642	3649	3717	3690	3663		3666	33
300	3735	3690	3645	3685	3645	3663	3694	3708	3588			3672	43
400	3626	3619	3593	3666	3683	3676	3649	3717				3653	39
500	3568	3506	3556	3445	3573	3586	3581					3545	51
600	3420	3438	3426	3436	3422	3432						3429	7
700	3460	3434	3482	3404	3448							3445	29
800	2941	2985	2930	2909								2941	32
900	2586	2980	2990									2852	230
1000	2695	3030										2862	236
1100	2736											2736	n/a

**Table 4:** Measured cube compressive strength and UPV data at 28 days.

Cube	1	2	3	4	5	6	Average
Compressive strength (MPa)	27.3	25.0	26.0	24.8	27.6	31.3	27.0
UPV (m/s)	3680	3492	3595	3459	3748	4011	3664

**Table 5:** Compressive strength and UPV data at 28 days.

Modified UPV and Compressive Strength	Plain	C60D12	C60D20	C60D32	C20D20	C40D20	C60D20	C60D12
UPV (m/s)	3639	3648	3717	3666	3656	3709	3717	3720
Compressive strength (MPa)	24.4	24.5	25.2	24.6	24.5	25.1	25.2	25.2

**Table 6:** Frequency and relative frequency for modified UPV.

Bins Array	Frequency and Relative Frequency											
	C20D20		C40D20		C60D20		C80D20		C60D12		C60D32	
(1400,1600]	1	1.5	0	0	0	0	0	0	0	0	0	0
(1600,1800]	0	0	0	0	0	0	0	0	0	0	0	0
(1800,2000]	0	0	0	0	0	0	0	0	0	0	0	0
(2000,2200]	1	1.5	0	0	0	0	0	0	0	0	0	0
(2200,2400]	4	6	0	0	0	0	0	0	0	0	0	0
(2400,2600]	1	1.5	0	0	0	0	0	0	0	0	1	1.5
(2600,2800]	2	3	0	0	0	0	0	0	0	0	0	0
(2800,3000]	2	3	1	1.5	1	1.5	0	0	0	0	2	3
(3000,3200]	2	3	0	0	0	0	0	0	0	0	0	0
(3200,3400]	8	12.1	1	1.5	2	3	1	1.5	1	1.5	7	10.6
(3400,3600]	7	10.6	10	15.1	7	10.6	5	7.5	8	12.1	3	4.5
(3600,3800]	38	57.5	54	81.8	56	84.8	60	90.9	57	86.3	53	80.3

## Data Availability

The data used to support the findings of this study are included in the article.
